# Case report: dengue fever associated acute macular neuroretinopathy

**DOI:** 10.3389/fmed.2024.1379429

**Published:** 2024-03-22

**Authors:** How-Chen Wang, Chia-Ching Lin, Chia-Hsin Chang, Jih-Jin Tsai

**Affiliations:** ^1^School of Medicine, College of Medicine, Kaohsiung Medical University, Kaohsiung, Taiwan; ^2^Department of Ophthalmology, Kaohsiung Medical University Hospital, Kaohsiung, Taiwan; ^3^Tropical Medicine Center, Kaohsiung Medical University Hospital, Kaohsiung, Taiwan; ^4^Division of Infectious Diseases, Department of Internal Medicine, Kaohsiung Medical University Hospital, Kaohsiung, Taiwan

**Keywords:** dengue fever, ocular manifestations, acute macular neuroretinopathy (AMN), maculopathy, DENV-1, case report

## Abstract

Dengue fever (DF), which is caused by the dengue virus (DENV) and transmitted through Aedes mosquitoes, is well recognized for its systemic manifestations, with its ocular involvement gaining recent attention. We present a case of a 41-year-old Taiwanese female who developed acute macular neuroretinopathy (AMN) following a DF diagnosis related to DENV-1, emphasizing the need for awareness of this complication. The patient, with a history of completely resolved optic neuritis (ON) and comorbidities, experienced blurred vision on day 10 after the onset of DF. The ophthalmic examination revealed macular edema, ellipsoid zone (EZ) infiltration, and choriocapillaris involvement. Despite pulse therapy with corticosteroids, visual disturbances persisted, highlighting the challenge of managing ocular complications. Ocular manifestations in DF include hemorrhages, inflammation, and vascular complications. DF-associated AMN, a rare presentation, poses challenges in diagnosis and treatment response evaluation. While most patients recover spontaneously, some face persistent visual impairment, especially with AMN. Our case emphasizes the importance of recognizing ocular complications in DF, necessitating a multidisciplinary approach for optimal management and further research to delineate treatment strategies and outcomes.

## Introduction

Dengue fever (DF) is an arbovirus disease transmitted mainly through Aedes mosquitoes. According to the 2009 World Health Organization's dengue classification, dengue virus (DENV) infection can result in either non-severe or severe disease. The non-severe form typically presents as a flu-like illness characterized by high fever, severe headache, myalgia, arthralgia, nausea, vomiting, and rashes. On the other hand, severe dengue can lead to plasma leakage, causing dengue shock syndrome (DSS), bleeding, or severe involvement of other organ systems (1).

Despite the well-documented systemic manifestations of DF, ocular involvement has gained recognition more recently, however, it remains a component often overlooked by primary care physicians. Through hemorrhagic, inflammatory, or vascular mechanisms (2), ocular involvement presents as sudden visual acuity impairment or central scotoma that often coincidences with platelet count nadir (3, 4). Common complications of the anterior segment include subconjunctival hemorrhage and uveitis, while posterior segment complications are commonly maculopathy or optic neuropathy (5). We hereby present a case of developing acute macular neuroretinopathy (AMN) shortly after the diagnosis of DF and aim to drive attention to such complications.

## Case report

A 41-year-old Taiwanese female contracted DENV-1 infection during the 2023 endemic in Taiwan and was confirmed by positive results of dengue non-structural protein 1 (NS1) antigen assay and polymerase chain reaction (PCR) examination. The platelet count reached the nadir at 102,000 cells/L on day 7 after the onset of DF (Post Symptom Onset Day 7, PSO 7). She had a history of optic neuritis (ON) in the left eye (oculus sinister, OS) more than 10 years ago. This time, she suffered from sudden onset blurred vision OS on PSO 10 with a white blood cell count of 7,600/μl and a platelet count of 272,000 cells/L. There were no pain or limitations in the eye movement.

**Figure 1:**
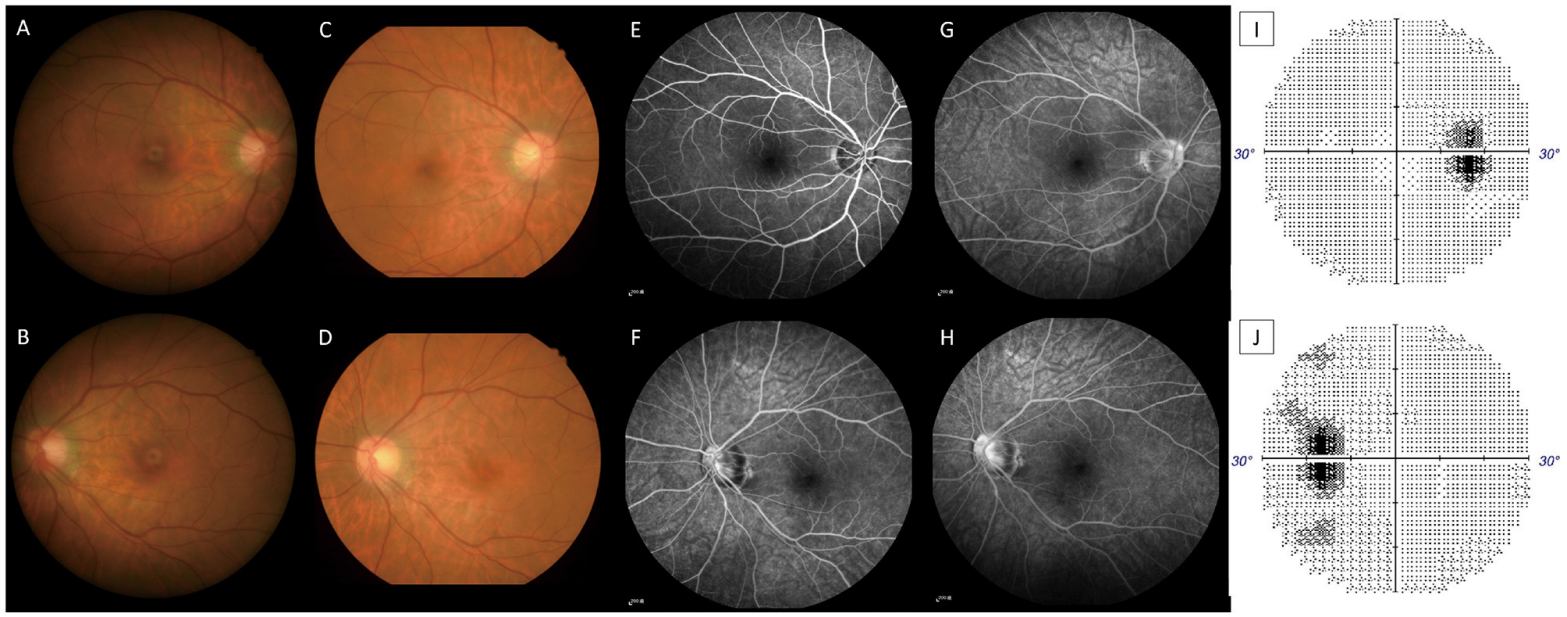
Color fundus on PSO 11 OD **(A)**, OS **(B)**, PSO 16 OD **(C)**, and OS **(D)**. The nasal disc hyperemia and narrower disc cupping (OU) are more prevalent when compared with the resolution stage on PSO 16. Fluorescent angiography on PSO 16; OD at 0'51” **(E)**, OS at 2'05”**(F)**, OD at 6'33” **(G)**, and OS at 5'18” **(H)** showed late vascular leakage from the retinal vessels and disc. Automated perimetry on PSO 17 OD **(I)**, OS **(J)** showed enlarged blind spot OU. All image indicates signs of retinal vasculitis and disc inflammation (optic papillitis).

**Figure 2:**
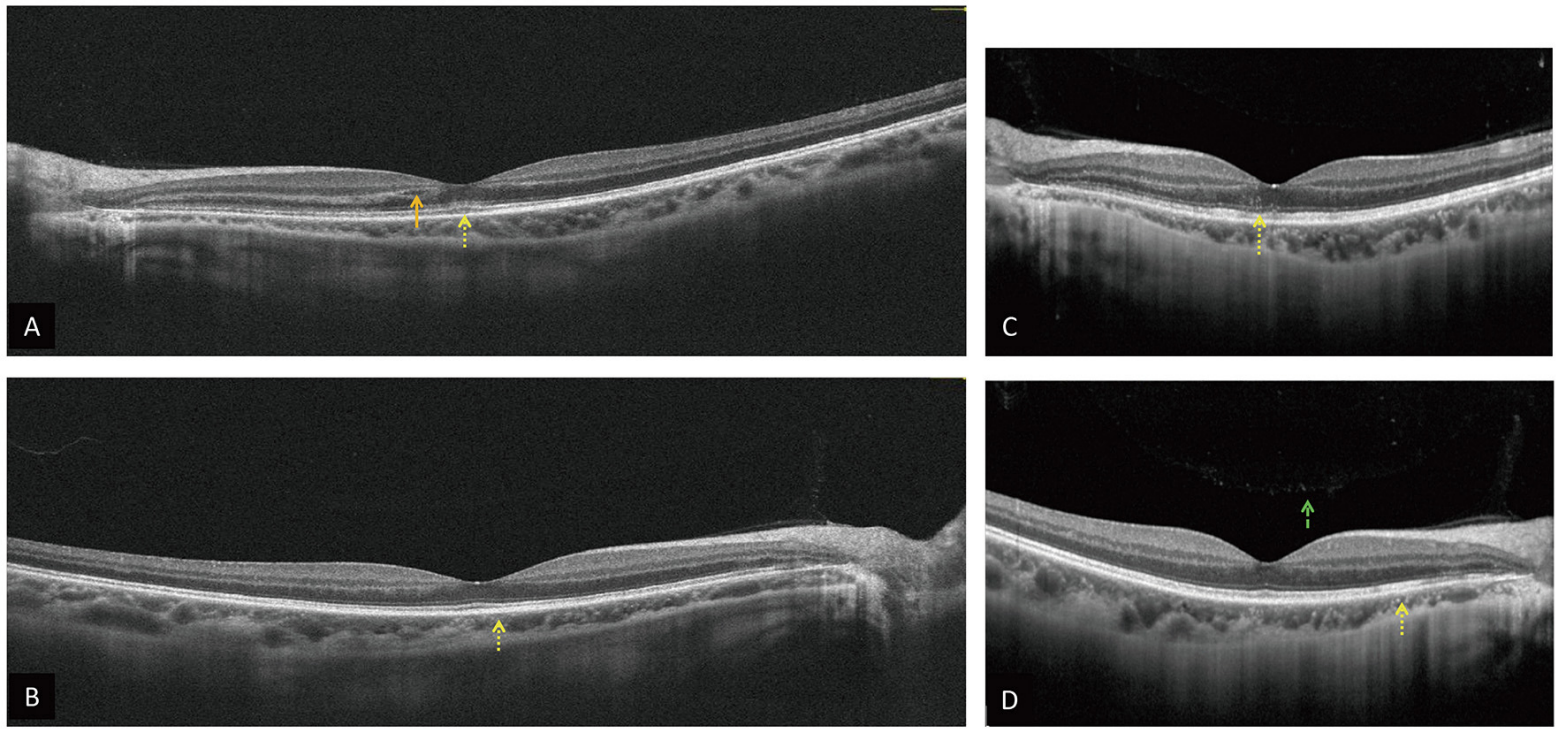
OCT on PSO 11 OD **(A)**, OS **(B)** reveals intraretinal fluid (orange arrow), hyperreflective materials in the superficial, deep retina, and choroid OU, especially at the EZ OS (yellow dot arrow). OCT on PSO 16 OD **(C)**, OS **(D)** show similar hyperreflective materials OU (yellow dot arrow) and vitreous hyperreflective foci OD [**(D)**, green dash arrow].

**Figure 3:**
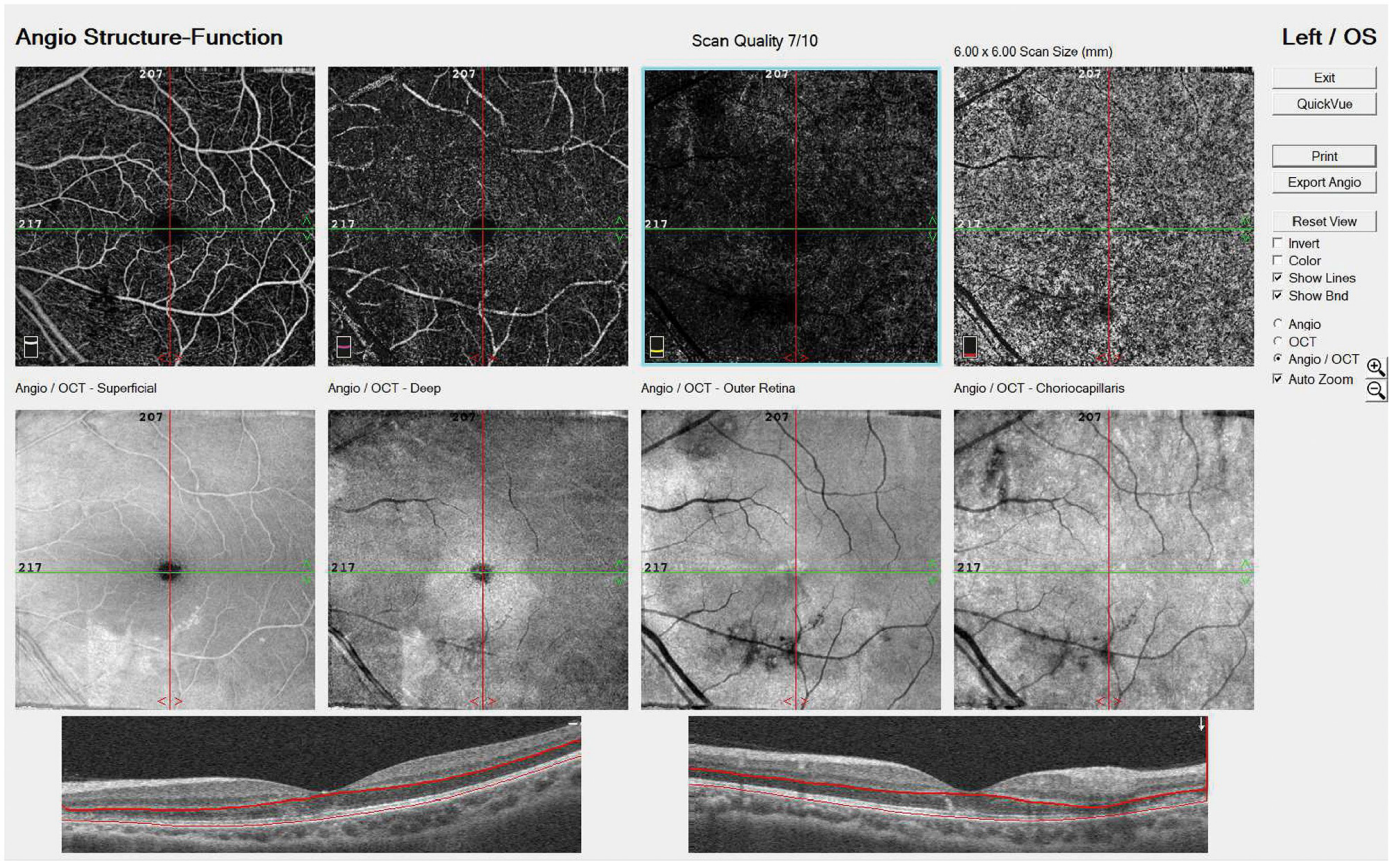
EN FACE OS on PSO 13 showed vascular damage from the superficial retina down to choriocapillaris.

The ophthalmic examination on PSO 11 revealed her best corrected visual acuity (BCVA) being 1.0 per glass (PG) right eye (oculus dexter, OD) and 0.05 PG OS. There was minimal relative afferent pupillary defect OS. The color plate test was 11/15 OD and 5/15 OS. Intraocular pressure (IOP) was 8.8 millimeters of mercury (mmHg) OD and 9.5 mmHg OS. The slit lamp showed no remarkable findings. Fundus examination (Figures 1A, B) and optical coherence tomography (OCT, Figures 2A, B, 3) showed macular edema, disc hyperemia on both eyes (Oculus Uterque, OU) with ellipsoid zone (EZ) infiltration, and probably involving the choriocapillaris OS. Magnetic resonance imaging (MRI) of the head was also performed, but no evidence of active ON is present (Supplementary Figure 1). She was then diagnosed with dengue maculopathy OU, with OS more severe than OD.

Systemic survey accidentally found type 2 diabetes mellitus, hypertension, and hypertriglyceridemia, while excluding human immunodeficiency virus (HIV) infection and syphilis, and yielded a negative result in antinuclear antibodies, anti-phospholipid autoantibodies, and the connective tissue disease screening panel (Anti-CTD screen).

She received pulse therapy with intravenous 250 mg of methylprednisolone four times daily for 1 day, followed by intravenous dexamethasone 5 mg once, along with oral aspirin 100 mg daily, and topical prednisolone.

However, blurred vision persisted, and a new central scotoma OS was noted on PSO 15. A follow-up examination found worse BCVA (0.6 OD and 0.0125 OS) and new mild vitreous haze (2+) OS. Therefore, oral prednisolone 40 mg daily was given for 1 week and was tapered down gradually over 2 months. Color fundus (Figures 1C, D) on PSO 16 found macular and perimacular edema. Fluorescent angiography (Figures 1E–H) and OCT (Figures 2C, D) on PSO 16 showed capillary leakage, vitreous opacity OS, and late staining at disc OU. Visual field examination (Figures 1I, J) on PSO 17 showed an enlarged blind spot OS with a mean deviation (MD) of −4.86 decibels (dB). All evidence, including vitritis, small vessel vasculitis-related maculopathy, and optic disc inflammation, are consistent with the diagnosis of AMN.

**Figure 4:**
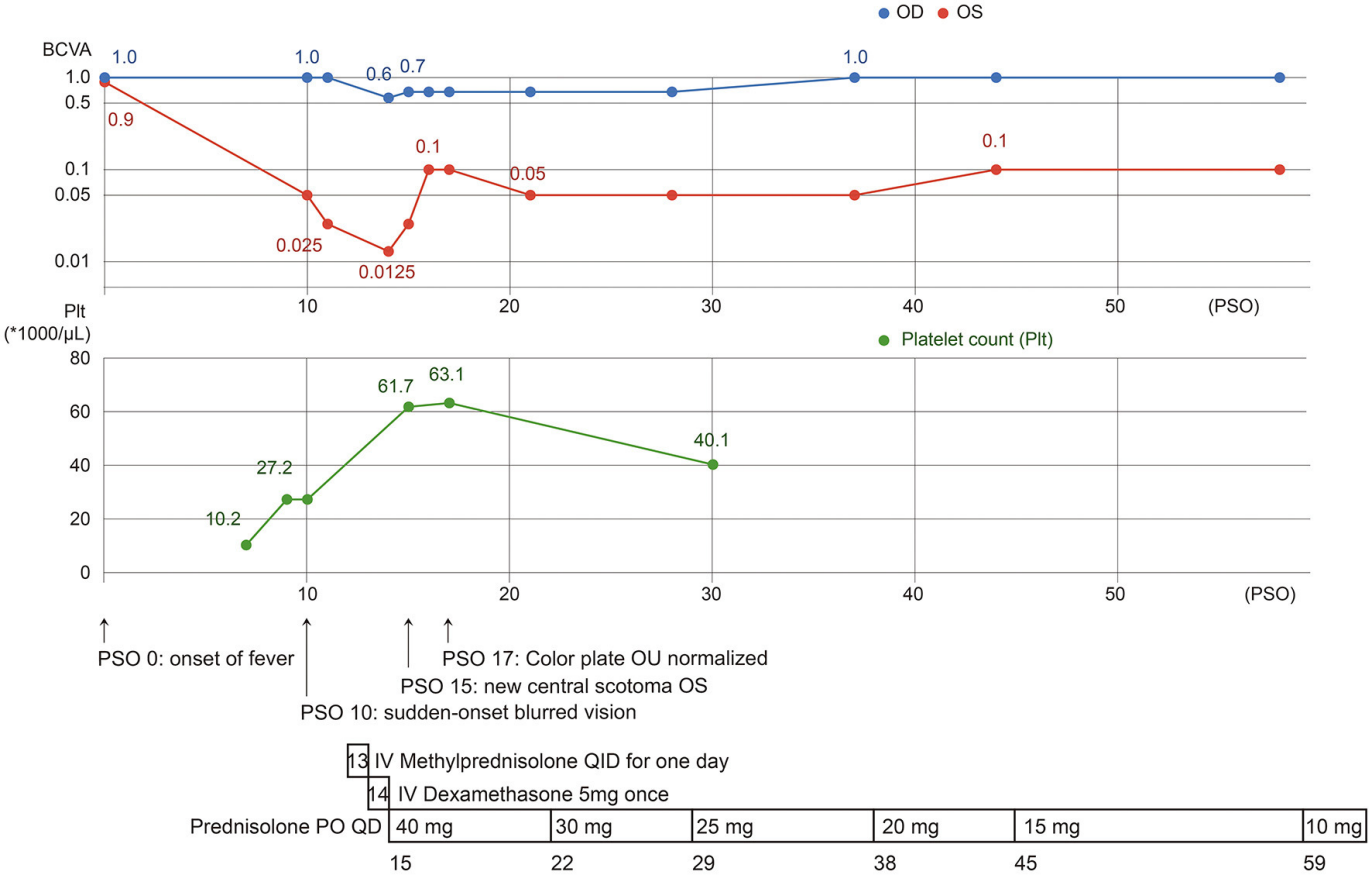
The clinical course of our case, with presentation of visual impairment since PSO 10, 3 days after the platelet count nadir. After aggressive treatment with topical, intravenous, and systemic corticosteroids, visual acuity improved but was limited in OS.

With treatment, the color plate test soon normalized, no more vitreous cell was found, and BCVA gradually improved within a week. Follow-up BCVA was 0.7 OD and 0.05 OS on PSO 29 and 1.0 OD and 0.1 OS on PSO 38. During her latest follow-up, color fundus and OCT showed less hyper-reflective material with residual EZ disruption and the BCVA improved to 0.16 OS. The patient is currently on low-dose oral corticosteroids with a maintenance dosage of 5 mg on PSO 59 (Figure 4).

## Discussion

Our patient was a victim of DENV-1 infection, presenting blurred vision and central scotoma on PSO 10, and AMN was finally diagnosed. She was treated with systemic and topical corticosteroids. Visual acuity and scotoma gradually improved after treatment but were limited (Figure 4).

To our knowledge, the last documentation on the ocular manifestation of DF in Taiwan can be traced back to 34 years ago (6, 7). However, the diagnostic approach of DF, novel ophthalmologic instruments, such as OCT, and many reviews on this topic have brought new perspectives on the ocular manifestations of DF. Over the past decade, as the prevalence of DF grew, there has been a booming investigation into the ocular involvement of DF worldwide. A big scale of DF outbreak occurred in Taiwan in 2023. Therefore, we wish to draw attention to this increasingly prevalent complication.

Ocular involvement of DF occurs in approximately 10% of hospitalized DF patients (8). Although the pathophysiology is still unclear, it is postulated that DF involves the eye through hemorrhagic, inflammatory, and vascular etiologies (2). Hemorrhages due to thrombocytopenia can occur in the conjunctiva, subconjunctiva, or retina, typically in the acute stage of infection within 24 h after nadir of platelet count (3, 4). Inflammatory manifestations are postulated to be a humeral or cellular reaction to viral antigens, cross-reaction, blood-eye barrier break, or immune complex deposition, presenting as uveitis of any segment, keratitis, acute angle-closure glaucoma, or maculopathy. Immune-mediated manifestations are often delayed by approximately a week (9–11). Immune complex disposition, endothelial disruption, and coagulative status secondary to the immune process may lead to retinal vasculitis and subsequent retinal vascular occlusion (11).

Dengue maculopathy usually occurs bilaterally with an asymmetric presentation on average PSO 7. Three phenotypes of maculopathy are proposed with OCT imaging, namely diffused retinal thickening, cystoid macular edema, and foveolitis, with the latter having a poor visual prognosis (12). While most patients present with poor visual acuity or central scotoma, some remain asymptomatic (8).

Conversely, uveitis exhibits divergent presentation and may affect any ocular segments. Anterior uveitis is typically non-granulomatous and either unilateral or bilateral (13). Intermediate uveitis is relatively infrequent. Posterior uveitis, a common complication, manifests as retinal vasculitis, exudative retinal detachment, multifocal chorioretinitis, acute posterior multifocal placoid pigment epitheliopathy, choroiditis, acute zonal occult outer retinopathy, or neuroretinitis (11, 14).

Ocular manifestations of DF frequently recover spontaneously without treatment. The management strategies for visual impairment or bilateral involvement include the use of topical corticosteroid eye drops and oral or intravenous corticosteroids. In refractory cases, corticosteroid pulse therapy and immunoglobulin administration can be considered (5). Furthermore, systemic immunosuppressant offers a viable steroid-free choice (15). However, determining the optimal treatment or evaluating the effect of these interventions can be challenging and requires rigorous controlled studies, given that spontaneous recovery can happen as a natural course.

The prognosis of DF ocular involvement is often good, and most patients experience complete recovery in weeks to months spontaneously or after treatment. However, vasculitis, dengue maculopathy, and optic neuropathy can be vision-threatening (2), and there are also cases with persistent scotomas and impaired visual function, especially those with AMN (16).

Several points require discussion in our case. To begin with, our patient had a history of ON OS over a decade ago. Although she achieved resolve of BCVA 0.9 OS then, ON may bring about unnoticed sequela or predisposition to ocular complications. ON is a relatively rare complication of DF, but onset and relapse due to infections can be possible (17, 18). Although electrodiagnostic examinations, such as visual evoked potential (VEP), were not performed on our patient, she had no typical symptoms, such as retro-orbital pain, and MRI and fundus examination found neither a sequela of past ON nor evidence of acute ON OS. As lack of pain is almost indicative of intracranial ON (19, 20), which is not found in our patient's MRI, and MRI images are sensitive for ON (21), we concluded that optic neuritis may not be the diagnosis for our patient. Moreover, she was diagnosed with comorbidities, including type 2 diabetes mellitus, hypertension, and hypertriglyceridemia, during DF infection. Whether these factors predisposed the risk of ocular complications needed to be further discussed (22). Fourth, she was treated with pulse therapy for 1 day, intravenous corticosteroid for 1 day, and oral and topical corticosteroid tapered down over 2 months. After the treatment, the right eye resolved completely, but improvement in the left eye was limited. In previous studies, treatment with oral prednisolone of 1 mg/kg/day for 3 days or intravenous methylprednisolone of 1 g/d for 3 days is both commonly used choices of regimen regarding the severity of presentation and tolerance to the medication. An improvement in the condition may take up to 3 months, and patients with AMN may have poorer outcomes (16). Whether corticosteroid therapy brings about better outcomes needs further controlled study. Finally, while our patient is diagnosed with AMN, a possible differential diagnosis of paracentral acute middle maculopathy (PAMM) is considered. PAMM is the OCT finding appearing as an inner nuclear layer (INL) hyperreflective band, presenting clinically as transient to permanent paracentral scotoma. As PAMM resolves, thinning of INL may be seen on OCT. In PAMM, OCT angiography may present deep retinal capillary plexus (DCP) hypoperfusion, while flow loss of choriocapillaris or DCP may be found in AMN (23). In our case, both INL reflective band and flow change in the vascular plexus are vague and PAMM is considered.

In conclusion, DF may bring about ocular manifestations, commonly blurred vision and central scotoma. Most patients have good visual outcomes after treatment, while some may suffer from persistent sequelae. Our patient suffered from visual disturbance resulting from AMN on PSO 10 after DENV-1 infection, received systemic and topical corticosteroid therapy, and had a complete remission OD but persistent poor vision OS.

## Data availability statement

The original contributions presented in the study are included in the article/Supplementary material, further inquiries can be directed to the corresponding author.

## Ethics statement

The studies involving humans were approved by Institutional Review Board of Kaohsiung Medical University Hospital. The studies were conducted in accordance with the local legislation and institutional requirements. The participants provided their written informed consent to participate in this study. Written informed consent was obtained from the individual(s) for the publication of this case report, and for any potentially identifiable images or data included in this article.

## Author contributions

H-CW: Conceptualization, Data curation, Methodology, Visualization, Writing—original draft, Writing—review & editing. C-CL: Conceptualization, Data curation, Investigation, Methodology, Resources, Supervision, Validation, Writing—original draft, Writing—review & editing. C-HC: Conceptualization, Data curation, Methodology, Writing—original draft, Writing—review & editing. J-JT: Conceptualization, Data curation, Formal analysis, Funding acquisition, Investigation, Methodology, Project administration, Resources, Supervision, Validation, Writing—original draft, Writing—review & editing.
