# Supplementary figures and images for: Case report: dengue fever associated acute macular neuroretinopathy
Source: Front Med (Lausanne). 2024 Mar 22;11:1379429. doi: 10.3389/fmed.2024.1379429 (PMC10995331; doi:10.3389/fmed.2024.1379429)

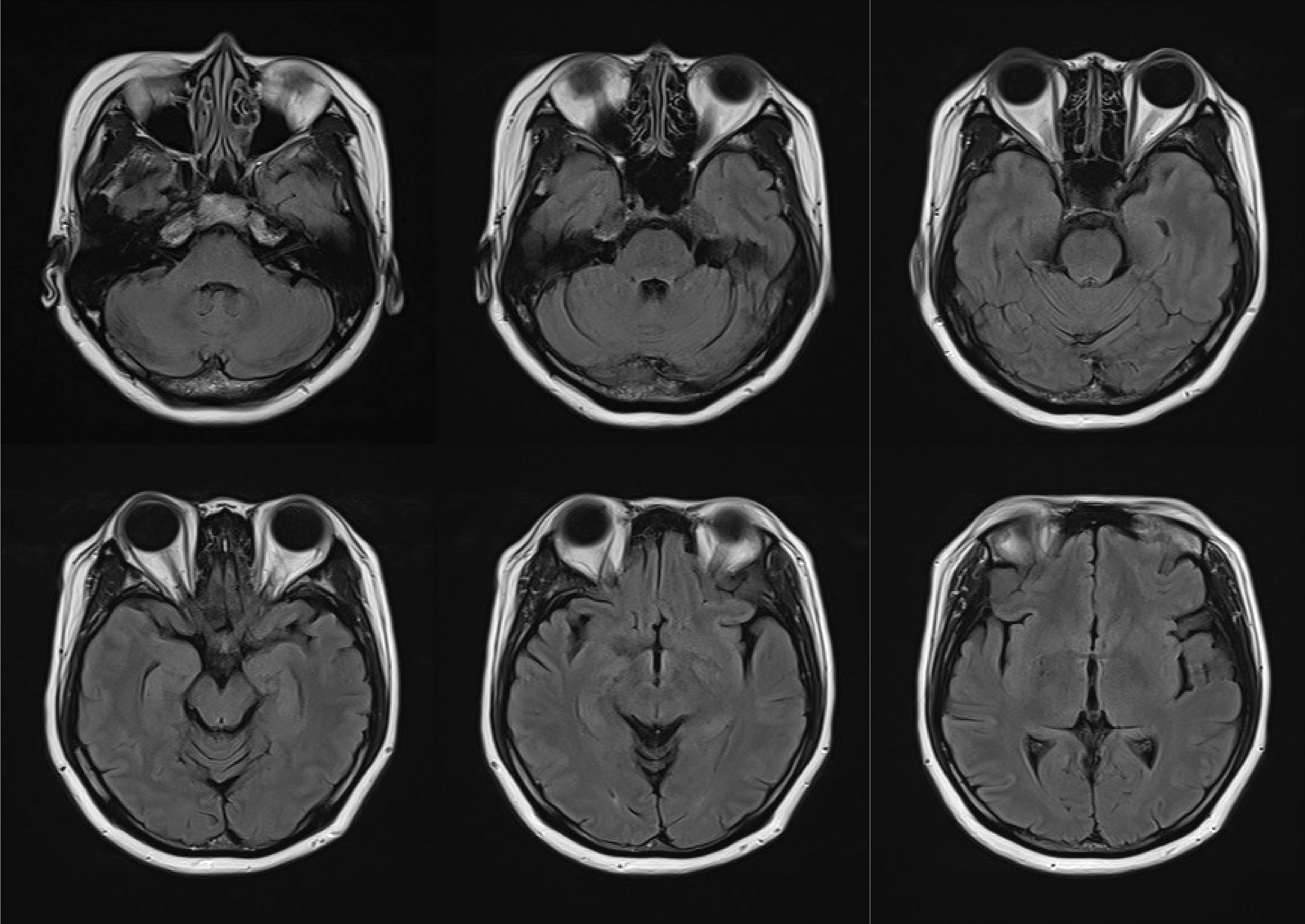

Supplement: Supplementary file 1 [file Image_1.JPEG]
